# Hypertonic Saline for Moderate Traumatic Brain Injury: A Scoping Review of Impact on Neurological Deterioration

**DOI:** 10.1089/neur.2020.0056

**Published:** 2020-12-15

**Authors:** Heather Rossong, Mohammed Hasen, Bilal Ahmed, Frederick A. Zeiler, Perry Dhaliwal

**Affiliations:** ^1^ Undergraduate Medicine, University of Manitoba, Winnipeg, Manitoba, Canada.; ^2^ Section of Neurosurgery, Department of Surgery, University of Manitoba, Winnipeg, Manitoba, Canada.; ^3^ Department of Human Anatomy and Cell Science, Rady Faculty of Health Sciences, University of Manitoba, Winnipeg, Manitoba, Canada.; ^4^ Biomedical Engineering, Price Faculty of Engineering, University of Manitoba, Winnipeg, Manitoba, Canada.; ^5^ Centre on Aging, University of Manitoba, Winnipeg, Manitoba, Canada.; ^6^ Division of Anaesthesia, Department of Medicine, Addenbrooke's Hospital, University of Cambridge, Cambridge, United Kingdom.

**Keywords:** hypertonic saline, outcome, moderate traumatic brain injury, scoping review, TBI, transition in care

## Abstract

Hypertonic saline (HTS) is a commonly administered agent for intracranial pressure (ICP) control in traumatic brain injury (TBI). The literature on its use is mainly in moderate/severe TBI where invasive ICP monitoring is present. The role of HTS in patients with moderate TBI (mTBI) outside of the intensive care unit (ICU) setting remains unclear. The goal of this scoping review was to provide an overview of the available literature on HTS administration in patients with mTBI without ICP monitoring, assessing its impact on outcome and transitions in care.

We performed a scoping systematic review of the literature of MEDLINE, Embase, Scopus, BIOSIS, and the Cochrane Databases from inception to July 31, 2020. We searched for those published articles documenting the administration of HTS in patients with mTBI with recorded functional outcome or transitions in hospital care. A two-step review process was conducted in accordance with methodology outlined in the *Cochrane Handbook for Systematic Reviews of Interventions*. There were many studies with combined moderate/severe TBI populations. However, most failed to document subgroup analysis for patients with mTBI. Our search strategy identified only one study that documented the administration of HTS in mTBI in which subgroup analysis for mTBI and outcomes were provided. This retrospective cohort study assessed patients with mTBI who did/did not receive prophylactic HTS, finding that those not receiving HTS demonstrated a deterioration in Glasgow Coma Scale (GCS) score in the first 48 h. However, the HTS group did demonstrate a trend to longer hospital stay and pneumonia. Our scoping review identified a significant gap in knowledge surrounding the use of HTS for patients with mTBI without invasive ICP monitoring. The limited identified literature suggests prophylactic administration prevents clinical deterioration, although this is based on a single study with data available for mTBI sub-analysis. Further studies on HTS in non-monitored patients with mTBI are required.

## Introduction

Cerebral edema is a major contributing factor of morbidity and mortality in patients with traumatic brain injury (TBI).^1^ An increase in intracranial pressure (ICP) results in decreased cerebral perfusion pressure (CPP) and cerebral blood flow (CBF). This ultimately contributes to cerebral hypoxia, ischemia, herniation, and death.^2^ To combat the deleterious effects of progressive cerebral edema in moderate/severe TBI, various guideline-based therapeutics have been developed. One such cornerstone of TBI therapeutics is the use of hyperosmolar/hypertonic agents. Examples of hyperosmolar therapy include solutions such as mannitol and hypertonic saline (HTS). The goal of this therapy in TBI is to decrease cerebral edema and prevent/reduce secondary brain injury.

HTS is an osmotherapeutic agent that is being used in practice as an alternative to mannitol in the treatment of cerebral edema, typically guided by continuous invasive ICP monitoring. The literature suggests a possible benefit of HTS over mannitol due to its lower blood–brain barrier (BBB) permeability and superior side-effect profile, especially in regard to renal failure.^3^ However, this is not a consistent finding. Review of the literature shows encouraging results in animal studies, but human studies are limited and there remain few studies examining the clinical outcomes of HTS after TBI. As per the Brain Trauma Foundation's (BTF's) *Guidelines for the Management of Severe Traumatic Brain Injury*, there exists a lack of ample evidence for the clinical outcomes of hyperosmolar therapy.^4^ Further, the predominance of literature focuses on severe TBI (sTBI; Glasgow Coma Scale [GCS] score <8), whereas milder forms of TBI remain relatively unstudied.

In particular, the role of HTS administration in patients with moderate TBI (mTBI), defined as a GCS score of 9–12, remains unclear. Many patients with mTBI do not require invasive ICP monitoring, yet still are at risk for neurological deterioration secondary to progressive cerebral edema. Thus, there may be a role for HTS administration in this cohort, in the absence of invasive ICP monitoring, to avoid progressive cerebral edema, subsequent neurological deterioration, and requirement for intensive care unit (ICU) level intervention. The goal of this systematically conducted scoping review was to outline the available literature on the use of HTS in patients with mTBI. Our primary objective was to evaluate whether the use of HTS (infusion or boluses) to maintain a state of hypernatremia reduces the frequency of neurological deterioration by >2 GCS points in patients with moderate TBI compared with those patients undergoing usual treatment. Whereas our secondary objectives of this review were to: 1) evaluate whether maintenance of hypernatremia after mTBI reduces the rate of surgical intervention or mortality rate compared with patients undergoing usual treatment; 2) evaluate whether there are increased complication rates with the use of HTS in patients with mTBI compared with patients undergoing usual treatment; and 3) evaluate the length of stay in the hospital relative to patients undergoing usual treatment.

## Methods

This scoping review was conducted in a systematic fashion, in keeping with the methods and techniques outlined in the *Cochrane Handbook for Systematic Reviews of Interventions*.^5^ Results are reported in keeping with the Preferred Reporting Items for Systematic Review and Meta-Analysis (PRISMA).^6^ The PRISMA checklist can be found in Supplementary Table S1. The review question, search strategy, and outcomes of interest were defined by the supervisor (P.D.) and primary author (H.R.).

### Search question, population, and inclusion/exclusion criteria

The following question was posed for this systematically conducted scoping review: What is the effect of intravenous HTS administration versus no HTS administration, in preventing neurological deterioration in adult moderate TBI? The primary outcome of interest was whether HTS administration would prevent neurological deterioration during hospital stay. We defined neurological deterioration as a decrease in GCS score by 2 or more points. The secondary outcomes of interest were: 1) impact on need for surgical intervention for cerebral edema; 2) any complication associated with HTS administration; 3) length of hospital stay; and 4) transition in care to requiring more intensive management (i.e., transfer to higher care unit, need for intubation/mechanical ventilation).

#### Inclusion criteria

Criteria included an adult patient cohort, mTBI, intravenous HTS administration, comparator non-HTS mTBI group present, and neurological status recorded. Adult patients were defined as being 18 years or older, with mTBI (GCS score 9–12). The intervention group was defined as patients receiving intravenous administration of HTS as either a bolus or infusion in any concentration (3%, 7.5%, or 23.4% sodium chloride) for any duration, and the control group as those receiving a placebo, standard of care, or no therapy. Published clinical articles including observational studies (cohort studies, case control studies) and randomized controlled trials (RCTs) were included. For case-mix studies, >50% of the patients in the study must have been classified as having mTBI. Observational studies must have included more than 20 patients with mTBI.

#### Exclusion criteria

Criteria included pediatric populations (age less than 18 years), non-HTS hyperosmolar/hypertonic agent administered, sTBI, no comparator non-HTS group, and no documentation of outcome. Patients with ischemic or hemorrhagic stroke, or disseminated cerebral disorders (such as meningitis, encephalitis, or neoplasms) were also excluded. Further, studies evaluating patients with mTBI with isolated epidural or subdural hemorrhages and non-English studies were excluded.

### Search strategy

MEDLINE, EMBASE, Cochrane Central Register of Controlled Trials, Scopus Web of Science, and BIOSIS were searched from inception to July 31, 2020. The following terms related to HTS were searched: hyperosmolar therapy, hypertonic saline, hypertonic solutions, hypertonicity, saline solution, and sodium chloride. The following terms related to mTBI were searched: brain injury, traumatic brain injury, cerebral hemorrhage, craniocerebral trauma, traumatic subarachnoid hemorrhage, subarachnoid hemorrhage, brain trauma, craniocerebral injury, head injury, intracranial pressure, and neuro trauma. Supplementary Table S2 provides the search strategy for MEDLINE, with similar search strings used for the other listed databases.

### Study selection

Two authors (H.R. and B.A.) reviewed the titles and abstracts of all documents for eligibility criteria. Full text articles that met the eligibility criteria were reviewed in a second screening step. References of any considered articles were also reviewed to identify other potential studies relevant to this review. A third author (P.D.) acted as an arbitrator to settle discrepancies between reviewers. During the first and second filtering processes, articles pertaining to HTS administration in mixed mTBI/sTBI and unspecified patient cohorts, were pulled to determine if information regarding the mTBI cohort could be extracted.

### Data abstraction

Standardized data collection forms were developed and used to extract the necessary information. Data collected from each study included the objectives, type of study, population, inclusion criteria, exclusion criteria, sample size, control and treatment groups with basic demographics, study period, summarized treatment protocol, type of HTS, outcomes assessed, results based on outcomes assessed, and comments from the reviewing author.

### Bias assessment

Given the goal was to provide a comprehensive scoping review of the available literature, a formal bias analysis was not conducted.

### Statistical analysis

Given the limited and heterogeneous literature identified, and that the purpose was to provide a scoping overview of the literature, a formal meta-analysis was not performed.

## Results

### Search results and included study characteristics

**FIG. 1.**
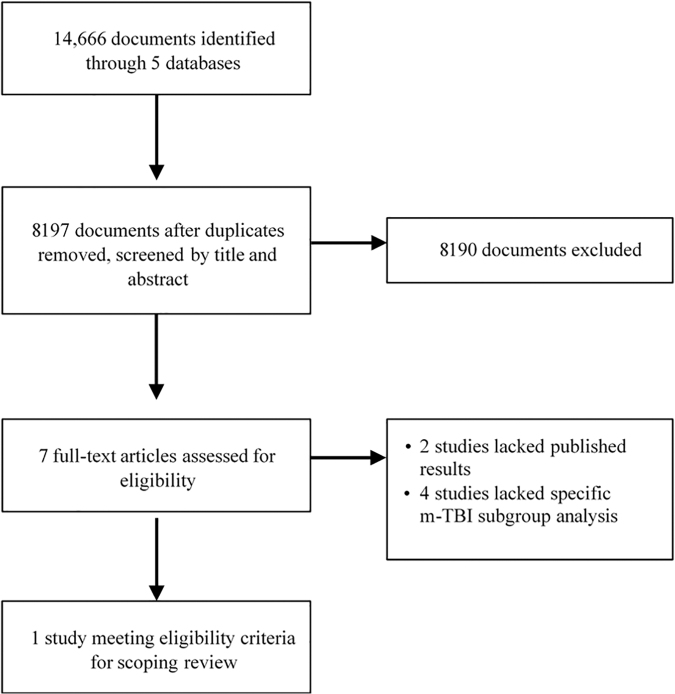
PRISMA flow chart of search results. PRISMA, Preferred Reporting Items for Systematic Reviews and Meta-Analyses; TBI, traumatic brain injury.

This search produced 14,666 documents, in which existed 4243 sets of multiple copies, leaving 8197 documents to review. After screening the titles and abstracts of all 8197 articles, 7 articles met the inclusion criteria and were reviewed in more depth. On secondary screening of the full 7 articles, 1 article was selected to be included in the final review. Figure 1 provides the PRISMA flow diagram of the search results. As mentioned above, studies were reviewed pertaining to HTS administration in mixed and unspecified TBI cohorts, to determine if specific comments regarding the mTBI cohort could be obtained.

All 7 studies used continuous HTS as the treatment arm except for 1 study that analyzed continuous infusion compared with bolus HTS.^7^ More than half of the articles (4/7, 57.1%) lacked a specific subgroup analysis for patients with mTBI compared with sTBI and were therefore excluded from this review.^7–10^ Two articles (2/7, 28.6%) lacked published results, with 1 only having an abstract available,^11^ whereas another was an RCT protocol with pending results.^12^ Subsequently, only 1 study with published results specifically analyzed the subgroup of mTBI patients and the use of HTS.^13^ This was also after checking the reference sections of the relevant articles for any missed articles, which failed to identify any.

### Final included study

Based on the above-mentioned filtering strategy, only 1 article met final inclusion in this review.^13^ This was a retrospective study of 205 adult patients admitted to the surgical/trauma ICU at Elmhurst Hospital, a Level 1 trauma center in New York City, between 2006 and 2012 with moderate to severe TBI. This study found that, for all patients with an initial GCS score >8 (*n* = 92), the GCS score remained the same in the HTS group at 48 h (12.8 ± 0.4 to 12.5 ± 0.5; *p* = 0.621) and worsened in the non-HTS group (13.6 ± 0.2 to 12.5 ± 0.5; *p* = 0.0468). Other scores were used including the Injury Severity Score (ISS) and Acute Physiology And Chronic Health Evaluation II (APACHE II) scores. ISS was worse in the non-HTS group (13 ± 1.4 vs. 18.3 ± 1.3; *p* = 0.026), whereas APACHE II scores were similar between the two groups (*p* = 0.285). However, the initial GCS scores for both the HTS and non-HTS groups in this subgroup analysis were 12.8 ± 0.4 and 13.6 ± 0.2, respectively. Therefore, this analysis may have included some patients with mild TBI (GCS score >13). Similarly, the HTS group consisted of younger patients (mean age 44.4 ± 3.3 years vs. 62 ± 2.7 years, *p* = 0.0001, respectively), and had a higher proportion of male patients. After correcting for initial GCS score, this study found a trend toward increased length of stay and pulmonary infections to be higher in the HTS cohorts. The conclusions of this study suggested that caution should be practiced when administering HTS to patients with TBI as neurological benefits are minimal but adverse events are considerable.

## Discussion

We conducted a comprehensive review of the literature, evaluating the available data on HTS administration for mTBI, and its impact on neurological status and outcomes. From the above outlined search strategy and results, we identified only one article meeting our inclusion/exclusion criteria. This was despite pulling articles with mixed mTBI/sTBI and undefined cohorts, to check if mTBI cohort data could be identified within the parent articles. Thus, overall, this was a negative systematic review. However, our findings, or lack thereof, do provide important points of discussion moving forward.

First, as displayed by the substantial lack of studies specifically analyzing the use of HTS in patients with mTBI, further research is needed in this patient population. HTS solutions are frequently used in patients with mTBI despite the clear lack of evidence for its efficacy and clinical outcomes. In theory, HTS primarily works to decrease cerebral edema, and subsequently ICP, by maintaining a state of hypernatremia and hyperosmolarity. The reflection coefficient of HTS is 1.0, meaning it does not cross the BBB and therefore drives free water from the brain into the vasculature.^14^ Reflecting on the larger body of available literature on HTS administration in sTBI (defined as a GCS score ≤8), important facts warrant highlighting. Recent meta-analysis of 12 RCTs with a total of 438 patients indicated no clear advantage of HTS over mannitol on mortality or neurological outcomes.^15^ Another meta-analysis of 11 RCTs displayed similar results,^16^ and supported recent TBI management guidelines, stating that the use of HTS in sTBI management lacks Level 1 evidence.

With that said, literature supports that HTS is associated with a faster resolution of ICP elevations. Yet, this advantage did not translate into morbidity and mortality benefits.^17^ In contrast, other recent systematic reviews and meta-analyses suggest that HTS does not exert a superior clinical effect over mannitol in terms of mortality and neurological outcomes.^18,19^ Such opposing findings in the literature are not uncommon among ICU-based therapeutics in TBI care. However, these benefits of HTS in the ICU-based management of sTBI don't necessarily trend to non-ICU management of mTBI cohorts, particularly, its use in the absence of objective invasive ICP monitoring, for the goal of preventing neurological deterioration in the mTBI patient population is unproven. The results of recent multi-center randomized trial efforts comparing ICP directed management in those with and without invasive ICP monitoring have displayed a potential role for ICP therapeutic strategies in non-monitored settings.^20^ However, this prior work primarily focused on the mTBI and sTBI populations requiring ICU-based management. The role of HTS to prevent deterioration of patients with mTBI, so that ICU level of care may be avoided, is unclear and unproven at this time, requiring much further study.

Second, the potential complication profile with HTS administration is something to consider in the mTBI population. HTS is not a treatment without associated risks. Suggested adverse events include renal insufficiency, fluid overload, electrolyte abnormalities including hypokalemia and hyperchloremic acidosis, rebound increased ICP, and potentially, osmotic demyelination syndrome (ODS).^21^ The single study evaluating HTS in mTBI in our review, comparing with a control group, found increased pulmonary complications. These concerns regarding HTS are echoed by recent evidence from the Collaborative European NeuroTrauma Effectiveness Research in TBI (CENTER-TBI) study, in which HTS use was found to be associated with increased risk of acute kidney injury in a large cohort of patients with TBI in the ICU.^22^

Further, recent data in multi-modal monitoring for patients with sTBI suggest the potential for HTS to induce worsening of cerebrovascular reactivity in those patients with normal autoregulatory status pre-HTS administration.^23^ Therefore, care should be taken when evaluating the need for HTS or other osmotherapeutic agents in patients with mTBI. Much further investigation in the complication profiles and acute physiological effects of HTS is required, as the above-mentioned studies only form a small literature body for complications.

Finally, regarding future work, this scoping review found a published prospective ICU study protocol that met the inclusion criteria.^12^ The COBI (COntinuous hyperosmolar therapy in traumatic Brain Injury patients) trial is a multi-center RCT of 370 patients in the ICU with TBI. This open-label, two-armed study randomizes patients to either: “standard care,” or continuous 20% HTS infusion plus standard care within the first 24 h post-trauma. The continuous HTS infusion is maintained for at least 48 h and continued for as long as needed. The primary outcome of this study is GCS score at 6 months and the study includes plans to sub-analyze patients with mTBI. This protocol has been approved and the results will aid in the question regarding the value of HTS in the mTBI patient population. The results of this ongoing work, in addition to some of the other future avenues of research regarding hyperosmotic/hyperosmolar agents in mTBI outlined below, will hopefully shed some light on the role of this drug in the non-ICU based management of mTBI.

### Limitations

Given the relative negative nature of the systematically performed scoping review, there are clear limitations regarding what can be gleamed from the above review. Clearly, having only one study fulfilling the inclusion/exclusion criteria, despite comprehensive and systematic literature searches of numerous databases, limits how much can be said. Similarly, observational studies analyzed in this scoping review inherently have their own limitations as observational studies cannot prove causation.^24^ It must be mentioned that this included study did have some patients with mild TBI in their “moderate TBI” cohorts, as seen in mean admission GCS scores slightly above 12. We acknowledge that this is not entirely in keeping with the above-defined inclusion criteria. However, this is the only study identified that focused on mTBI cohorts during HTS administration in which the vast majority were patients with mTBI. Hence, we elected to include it to highlight both the current literature and knowledge gap.

To date, there have been a number of reviews on the utility of HTS for patients with TBI in the ICU. Given this, we wanted not to reproduce already existing analyses of the literature, but to focus on one particular area of HTS administration for patients with mTBI. In the literature, the mTBI cohort is an often forgotten group of patients with TBI, with most work focused on mild TBI/concussion or advanced monitoring/intervention for the critically ill severe cohort. The mTBI group far outnumbers the severe cohort, yet has little focused research to support/refute interventions. We wished to simply provide an overview of the literature surrounding a commonly administered agent, HTS, and determine whether there is any documentation on the impact in the moderate cohort. As seen above, the literature on HTS was dominated by severe TBI cohorts, or mixed mTBI/sTBI groups from which it was not possible to extract information on the moderate group alone. This left us with a single article from which mTBI information could be extracted. Some may see this as a limitation. However, by highlighting the critical knowledge gap in the literature in mTBI, we believe that even though the review was essentially negative, we have been able to demonstrate an area for future critical research into the management of mTBI. Thus, the review should not be considered a total loss, but an example whereby a negative systematic search aids in the characterization of a knowledge gap, followed by an outline of crucial areas for future research.

### Future directions

A large gap in knowledge exists for the mTBI patient population when it comes to treating increased ICP and the utility of hyperosmolar/hyperosmotic agents. HTS is frequently used to lower cerebral edema but, as displayed in this review, the evidence is severely lacking. Further studies are needed to specifically analyze HTS in this subgroup of patients and evaluate its effect on clinical outcomes. Fortunately, through our review, we identified an ongoing prospective study on 20% HTS infusion in mTBI, and look forward to the results. This study alone will likely prove insufficient in answering all questions, supporting the need for additional prospective studies and analysis of large existing mTBI data sets co-registered to treatment information. One such existing data set optimally positioned for this is CENTER-TBI. With thousands of patients enrolled in the prospective observational study, there is a wealth of data available for analysis of the impact of various BTF guideline-based therapeutic strategies on patients with mTBI and outcomes, including long-term quality-of-life metrics. This is the focus of ongoing approved proposals within the CENTER-TBI group.

Aside from analysis of existing databases and development of pointed prospective randomized trials on the use of HTS in mTBI, work remains to be conducted to answer more fundamental questions into the physiological consequences of HTS, and other guideline therapeutics, in mTBI. There is a need for multi-modal cerebral physiological monitoring studies, where high-frequency physiological data are linked with temporally resolved treatment information. In the mTBI population, a focus on more non-invasive monitoring is required, and could potentially take the form of fixed wavelength near infrared spectroscopy (NIRS),^25,26^ robotic transcranial Doppler (rTCD),^27,28^ and non-invasive continuous arterial blood pressure (nABP) monitoring. Such physiological data can be used to provide information on pulsatile regional oxygen delivery,^26^ cerebral blood flow velocity,^28^ pulsatile cerebral blood volume,^28,29^ cerebrovascular reactivity,^30–33^ and compensatory reserve.^27^ Such real-time bedside metrics can be linked with treatment information, facilitating improved understanding of the temporal physiological effects of therapeutics, such as HTS, on various aspects of cerebral physiology.

Multi-center and international groups are exploring such work, including those in Europe^34–36^ and Canada.^37–39^ Our group in particular, the Winnipeg Acute TBI Laboratories, has recently led some of the preliminary work in this field, through the development of non-invasive techniques for continuous cerebrovascular reactivity assessments, and through interrogation of the impacts of HTS, sedation, and vasopressor agents on multi-modal cerebral physiological monitoring.^37–39^ Also, the CAnadian High-Resolution TBI (CAHR-TBI) research collaborative has been created to facilitate multi-center high-frequency physiological work in mTBI/sTBI, with specific mandates aimed at improving our understanding of the impact of guideline-based therapeutics on cerebral physiology.^37^ This future work requires multi-disciplinary expertise, with collaboration between clinicians, physiologists, engineers, data scientists, and population health experts.

## Conclusion

A systematically conducted scoping review of the literature was completed to analyze the use of HTS compared with standard therapy to treat cerebral edema in patients with mTBI (GCS score 9–12). Only one study with published results specifically analyzed the mTBI subgroup of patients and reported minimal neurological benefit in the HTS-treated patients over the non-HTS-treated patients. Such limited findings highlight a significant knowledge gap in mTBI care provision. Further research is needed to specifically analyze the use of HTS in the mTBI patient population to optimize the treatment of cerebral edema, decrease potential harms, and improve neurological outcomes.

## Supplementary Material

Supplemental data

Supplemental data

## Abbreviations Used

APACHE II: Acute Physiology And Chronic Health Evaluation II
BBB: blood–brain barrier
BTF: Brain Trauma Foundation
CAHR-TBI: CAnadian High-Resolution TBI
CBF: cerebral blood flow
CENTER-TBI: Collaborative European NeuroTrauma Effectiveness Research in TBI
COBI: COntinuous hyperosmolar therapy in traumatic Brain Injury patients
CPP: cerebral perfusion pressure
GCS: Glasgow Coma Scale
HTS: hypertonic saline
ICP: intracranial pressure
ICU: intensive care unit
ISS: Injury Severity Score
mTBI: moderate traumatic brain injury
nABP: non-invasive continuous arterial blood pressure
NIRS: near infrared spectroscopy
ODS: osmotic demyelination syndrome
RCT: randomized controlled trial
rTDC: robotic transcranial Doppler
sTBI: severe traumatic brain injury
TBI: traumatic brain injury
