# Supplementary material for: Hypertonic Saline for Moderate Traumatic Brain Injury: A Scoping Review of Impact on Neurological Deterioration
Source: Neurotrauma Rep. 2020 Dec 15;1(1):253–60. doi: 10.1089/neur.2020.0056 (PMC7769038; doi:10.1089/neur.2020.0056)
Supplement: Supplemental data [file Supp_TableS2.docx]

**Supplementary Table S2** : OVID/MEDLINE Search Strategy

1. Hyperosmolar therapy.mp.
2. Hypertonic Saline.mp.
3. Hypertonic solutions.mp.
4. Hypertonicity.mp.
5. Saline solution.mp.
6. Sodium chloride.mp.
7. 1 or 2 or 3 or 4 or 5 or 6
8. Brain injury.mp.
9. Traumatic brain injury.mp.
10. Cerebral hemorrhage.mp.
11. Craniocerebral trauma.mp.
12. Traumatic subarachnoid hemorrhage.mp.
13. Subarachnoid hemorrhage.mp.
14. Brain trauma.mp.
15. Craniocerebral injury.mp.
16. Head Injury.mp.
17. Intracranial pressure.mp.
18. Neuro trauma.mp.
19. 8 or 9 or 10 or 11 or 12 or 13 or 14 or 15 or 16 or 17 or 18
20. 7 and 19
